# Acute mania following COVID-19 in a woman with no past psychiatric history case report

**DOI:** 10.1186/s12888-022-04110-y

**Published:** 2022-07-20

**Authors:** Steven Sprenger, J. Pilar Bare, Rahul Kashyap, Luigi Cardella

**Affiliations:** Department of Psychiatry, Tristar Centennial Medical Center, HCA Healthcare, 2300 Patterson St, 37203 Nashville, TN USA

**Keywords:** COVID-19, Mania, Psychiatric, Mental health, Case report

## Abstract

**Background:**

The COVID-19 pandemic that began in late 2019 is caused by infection with the severe acute respiratory syndrome coronavirus-2. Since that time, many neuropsychiatric sequelae including psychosis, neurocognitive disorders, and mood disorders have been observed. The mechanism underlying these effects are currently unknown, however several mechanisms have been proposed.

**Case presentation:**

A 47-year-old woman with past medical history including hypertension and premenstrual syndrome but no psychiatric history presented to the psychiatric hospital with new onset mania. She had developed symptoms of COVID-19 and was later diagnosed with COVID pneumonia. During quarantine, she reported high levels of stress, grief, and anxiety. Seventeen days into her illness, she developed altered mental status, sleeplessness, elevated mood, talkativeness, and preoccupations. Her spouse was concerned for her safety and contacted emergency medical services who brought her to the psychiatric hospital. She had not slept for five days prior to her arrival and exhibited flight of ideas, talkativeness, and grandiose ideas. She reported a family history of bipolar disorder but no past manic or depressive episodes. She was diagnosed with acute mania and stabilized using antipsychotics, a mood stabilizer, and a short course of a benzodiazepine. Many of her symptoms improved, including her elevated mood, increased activity level, and flight of ideas though she continued to have decreased need for sleep as her benzodiazepine was tapered. She and her partner were agreeable to transitioning to outpatient care after her mood stabilized.

**Conclusions:**

This report emphasizes the link between COVID-19 and neuropsychiatric symptoms. Acute mania has no recognized association with COVID-19, but similar presentations have been reported. The patient’s age and time to onset of psychiatric symptoms is consistent with previous reports. Given the growing body of evidence, this association warrants further investigation. Severe acute respiratory syndrome coronavirus-2 causes systemic inflammation and has been shown to be neurotropic. In addition, patients undergoing quarantine experience anxiety related to the disease in addition to social isolation. Psychiatric practitioners should be aware of these effects and advocate for psychiatric evaluation following COVID-19 infection. Understanding the sequelae of infectious disease is crucial for responding to future pandemics.

## Background

The COVID-19 pandemic is caused by spread of the severe acute respiratory syndrome coronavirus-2, which was first observed in late 2019 [1, 2]. Since that time, a wide variety of neuropsychiatric sequelae have been observed [3, 4]. We are presenting a case of acute mania in a 47-year-old woman with no past psychiatric history starting 17 days after the onset of COVID-19. This case supports and association between COVID-19 infection and development of acute mania highlighting the importance of mental health in responding to the pandemic.

This is to our knowledge the first case of new onset acute mania in a female patient who was COVID-19 positive following outpatient treatment that did not include steroids. Other reported cases were all males from China, United Kingdom and New York, USA [5–7]. A woman with acute mania following quarantine has been reported, however she did not test positive for COVID-19 [8]. This case is also unique in that the assessment and care were conducted in a psychiatric hospital under close supervision.

## Case presentation

**Fig. 1:**
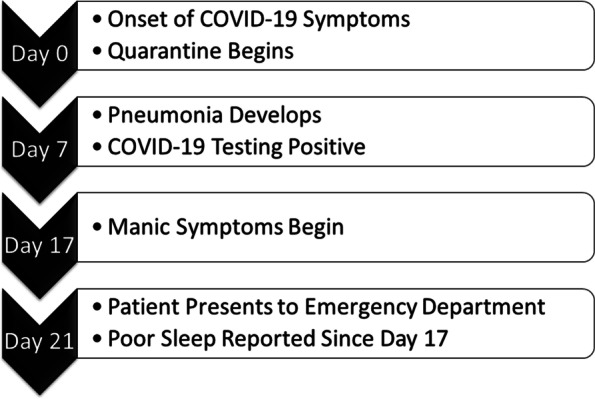
Timeline of patient presentation and progression starting at onset of COVID-19 symptoms (Time 0)

A 47-year-old woman with past medical history of hypertension and no psychiatric history presented to the psychiatric hospital with altered mental status. She was previously prescribed duloxetine for symptoms of premenstrual syndrome but otherwise denies a history of mood symptoms. Seventeen days before her arrival at the psychiatric hospital, she had developed symptoms suggestive of COVID-19 and began quarantining at the advice of her physician (Fig. 1). She was diagnosed as an outpatient with pneumonia on day seven and had a positive COVID-19 test at that time. She continued self-isolating and was managed conservatively as an outpatient. Symptomatic management included over the counter pain relievers and decongestants as needed, however the patient preferred not to take medications. Steroids were not prescribed. During self-isolation, she developed anxiety related to the pandemic becoming aware of several deaths in her social group secondary to COVID-19 and became anxious for her husband and herself. By day 17, she began exhibiting elevated mood, bizarre behavior, flight of ideas, talkativeness, sleeplessness, and grandiosity. On day 21, her spouse called emergency medical services because she was exhibiting bizarre behavior including infatuation with the number three, magical thinking, and uncharacteristic behaviors. For example, her husband was afraid when she reportedly held a knife and offered to make it disappear as a magic trick. She has slept very little since day 17 when her elevated mood began. She was brought to the emergency department by ambulance and was referred to the psychiatric hospital the following day. She has a family history of bipolar disorder but reports no previous manic or depressive episodes. Her spouse corroborated this, describing her mood historically as “rock solid.” The patient was employed and lived with her spouse.

On admission, the patient was evaluated for organic causes of her presentation. The medical staff determined there was no evidence of encephalopathy or altered mental status from toxic, metabolic, ischemic, infectious, or structural sources. Her vital signs were stable with a temperature of 98.8° F, heart rate of 90, blood pressure of 148/85 mmHg, oxygen saturation of 99% on room air, and respiratory rate of 16 breaths per minute. Laboratory evaluation included a complete metabolic panel and complete blood count which was noncontributory and did not reveal any potential cause for altered mental status. Notably, serology was negative for COVID-19 on arrival to the Emergency Department. Urine drug screening for alcohol and drugs of abuse was negative. The patient denied head trauma and no intracranial abnormalities were observed on imaging.

On arrival to the psychiatric hospital, an assessment was conducted. The patient demonstrated an expansive mood and exhibited flight of ideas, distractibility, increased goal directed behavior, restlessness, talkativeness, and grandiosity. Grandiose delusions were characterized by an elevated sense of self and the perception that she had been chosen to combat the pandemic. There were no obvious perceptual disturbances. There were no appreciable cognitive deficits. Her behavior included carrying papers on which she was planning a party with the intention to invite the entire world. The patient was euphoric, equating her stay to that of a five-star resort.” Despite having not slept in five days, the patient reported feeling energetic and feeling that she was “on top of the world.” She was diagnosed with acute mania and provided with pharmaceutical treatment in addition to group therapy. Delirious mania was considered, however no fluctuations in level of consciousness were observed, and the patient was neither disoriented nor confused. Choice of pharmacologic agent was made according to patient preference. Concerns were discussed regarding potential side effects and stigma regarding the use of lithium as a mood stabilizer. Quetiapine was chosen as an antipsychotic due to the presence of insomnia. A short course of benzodiazepine was also prescribed. The regimen utilized was extended release alprazolam 1 mg, quetiapine 100 mg, and lamotrigine 25 mg for mood. She reported improved sleep attributed to the initiation of these medications. Her quetiapine was titrated upwards by 100 mg each day, eventually reaching a total of 500 mg. Many of her symptoms improved, including her elevated mood, increased activity level, and flight of ideas, though decreased need for sleep returned somewhat as her benzodiazepine was tapered. She tolerated the medicines well and reported only drowsiness as a side effect. She and her partner were agreeable to transitioning to outpatient care after her mood stabilized. The length of stay was ten days from arrival to discharge. At the time of discharge, her mood was euthymic. She was no longer exhibiting flight of ideas, restlessness, or increased goal directed activity. Grandiose and bizarre behaviors had resolved, but overvalued ideas remained. She reported significant improvements in sleep.

## Discussion and conclusion

This case was a 47-year-old woman who presented with acute mania following infection with COVID-19. Possible explanations for the observed symptoms include neuropsychiatric effects of the virus, acute psychosocial stress during quarantine, or first presentation of a new disorder. Although there is not a recognized association between COVID-19 and new onset acute mania, previous reports have detailed similar presentations. A survey of 125 cases of COVID-19 demonstrated 21 new onset psychiatric disorders, one of which was acute mania [9]. Three case reports have detailed similar presentations of new onset of acute mania in patients without previous psychiatric history and ages ranging from 41 to 51 years old and COVID-19 infection preceding symptoms by between 10 and 21 days [5–7]. Extensive medical evaluation did not reveal alternative causes for the presentation, suggesting the symptoms may be secondary to the infection [7]. Precipitation of mania and concurrent delirium following infection of COVID-19 in a middle-age woman with no psychiatric history has also been observed [8].

One possible explanation for the development of mania is the interplay of acute psychological stress brought on by the pandemic, self-isolation, and uncertainty regarding physical illness. New onset acute mania has also been reported in a 32-year-old female patient with no previous psychiatric history. She was quarantined following an exposure, but subsequently tested negative for COVID-19. Her symptoms were hypothesized to have been triggered by acute stress [10]. The present case was characterized by a high degree of psychological stress during quarantine brought on by the awareness of COVID-19 deaths and anxiety regarding the health of her partner. The effect of these stressors on the observed symptoms must be considered.

Primary onset of a psychiatric disorder is also possible. The patient did report a family history of bipolar disorder in two first-degree relatives, however her age at presentation and lack of past psychiatric history makes this explanation less probable. Previous reports have all involved patients of similar age without past psychiatric history. It is unlikely that undiagnosed psychiatric disorders account for these presentations, suggesting a causative link between COVID-19 infection and new onset acute mania.

Several mechanisms have been proposed for the association between acute mania and COVID-19 infection. These include inflammation, viral encephalitis, and acute stress [5, 6, 10]. Inflammatory changes have been associated with manic episodes in bipolar patients [11]. SARS-CoV-2 has been shown to cause inflammation through a variety of mechanisms which has led to the hypothesis that neuropsychiatric symptoms may be caused by an inflammatory process [7, 12]. Another possible cause is direct infection of the nervous system. The presumed receptor for SARS-CoV-2 has been identified in brain tissue [13]. SARS-CoV-2 IgG was present in the cerebrospinal fluid of one patient who displayed mania following COVID-19 during his infection, however SARS-CoV-2 RNA was absent at the time that he developed manic symptoms [5]. Further investigation is needed to determine the causative etiology.

This case illustrates acute mania as a potential sequela of COVID-19 infection. To our knowledge, there have been four reported incidents of new onset mania following infection with COVID-19 in patients of a similar age. A single incidence of mania following quarantine in a COVID-19 negative patient has been observed in a younger patient. Care was administered in an inpatient psychiatric facility, allowing for assessment of response to treatment. A weakness of this report is the short duration of follow up and inability to indicate prognosis. Multicenter collaboration and pooling of patient data [14] is necessary to determine the frequency, prognosis, mechanism and possible treatments for this phenomenon.

Although the mechanism remains unknown, literature review suggests that new onset mania may be a potential sequelae of SARS-CoV-2 infection. Psychiatric screening for these disorders may play an important role in treatment. This case also highlights the relationship between COVID-19 and mental health. It is imperative that the mental health community support the broader public health effort to combat the pandemic.

## Data Availability

Not applicable.

## Abbreviations

COVID-19: Coronavirus disease 19
SARS CoV-2: Severe acute respiratory syndrome coronavirus-2
IgG: Immunoglobulin G
RNA: Ribonucleic acid
